# Toward a personalized closed-loop stimulation of the visual cortex: Advances and challenges

**DOI:** 10.3389/fncel.2022.1034270

**Published:** 2022-12-13

**Authors:** Fabrizio Grani, Cristina Soto-Sánchez, Antonio Fimia, Eduardo Fernández

**Affiliations:** ^1^Institute of Bioengineering, Universidad Miguel Hernández de Elche, Elche, Spain; ^2^Biomedical Research Networking Center in Bioengineering, Biomaterials and Nanomedicine (CIBER-BBN), Madrid, Spain; ^3^Departamento de Ciencia de Materiales, Óptica y Tecnología Electrónica, Universidad Miguel Hernández de Elche, Elche, Spain

**Keywords:** closed-loop stimulation, visual prostheses, neural interfaces, brain stimulation, local field potentials

## Abstract

Current cortical visual prosthesis approaches are primarily unidirectional and do not consider the feed-back circuits that exist in just about every part of the nervous system. Herein, we provide a brief overview of some recent developments for better controlling brain stimulation and present preliminary human data indicating that closed-loop strategies could considerably enhance the effectiveness, safety, and long-term stability of visual cortex stimulation. We propose that the development of improved closed-loop strategies may help to enhance our capacity to communicate with the brain.

## Introduction

Visual impairment has a profound impact on the lives of those who experience it (Bourne et al., 2017). Although some novel clinical approaches are becoming available (Higuchi et al., 2017; De Silva and Moore, 2022; Panikker et al., 2022; Van Gelder et al., 2022), unfortunately, there is no treatment for all causes of blindness (Fernandez, 2018; Fernandez et al., 2020). Thus, there are many blind patients for whom there is still no medical treatment. As a consequence of this growing and clearly unmet need, numerous groups worldwide are pursuing other approaches to provide at least a rudimentary sense of vision to the blind.

Visual prostheses are promising solutions to restore functional vision (i.e., visual percepts that could help blind people to recognize objects or to navigate in complex environments). Retinal prostheses are the most successful approach in this field to date (Nowik et al., 2020; Picaud and Sahel, 2020; Nanegrungsunk et al., 2022), but patients with severe retinal degeneration, glaucoma, or optic atrophy cannot get benefit from a retinal prosthesis. Therefore, there are compelling reasons for the development of alternative approaches that can bypass the retina to restore a functional sense of vision.

In this framework, although the optic nerve or lateral geniculate nucleus could be good targets (Nguyen et al., 2016; Gaillet et al., 2020; Borda et al., 2022; Rassia et al., 2022), several groups are trying to develop visual prostheses designed to directly stimulate the visual cortex (Lee et al., 2016; Beauchamp et al., 2020; Fernandez et al., 2021; Bosking et al., 2022). However, the biological and engineering problems for the success of cortical implants are much more complex than originally believed and involve, for example, long-term biocompatibility issues and challenges related to the encoding of visual information and the delivery of information to implants (Fernandez et al., 2020). In addition, we should be aware that the human brain is arguably one of the most complex systems in nature and that cortical stimulation should be safe, precise, and effective.

To achieve the ambitious objectives envisioned by cortical visual prostheses, we should be able to stimulate the occipital cortex in a way as similar as possible to the physiological response to visual stimuli, mimicking the human visual pathway (Nirenberg and Pandarinath, 2012; Qiao et al., 2019; Brackbill et al., 2020; Li et al., 2022; Price and Gavornik, 2022). In this framework, we should consider that closed-loop circuits exist in just about every part of the nervous system (Farkhondeh Tale Navi et al., 2022; Khodagholy et al., 2022). However, current cortical visual prosthesis approaches are primarily unidirectional and do not incorporate any adaptive system for the modulation of the electrical stimulation used to induce visual perception. Herein, we briefly introduce some recent advances for better control of brain stimulation and present preliminary human data suggesting that a closed-loop approach could significantly improve the performance, safety, and long-term stability of the stimulation of visual cortex neurons.

## Learning to control brain electrical stimulation

Electrical stimulation of the brain is the basis of many technologies for the restoration of sensory and motor functions. Brain stimulation has been used for reducing tremors in Parkinson’s patients, controlling epileptiform activity, and improving mood in patients with severe depression (Lozano et al., 2019). Additionally, it is now possible to create artificial sensations, with unprecedented resolution, *via* delivery of intracortical microstimulation (Fernandez et al., 2021; Fernandez, 2022; Fifer et al., 2022). However, most current brain stimulation approaches cannot flexibly control the patterns of activity because, for it to work, we need to know the activity of the neurons surrounding the electrodes and modulate the electrical stimulation in function of this neural activity.

Although stimulating electrodes allow control of the dynamics of populations of neurons, they do not provide insights into the electrophysiological activity of the neurons surrounding the electrodes. Thus, a critical step in the development of closed-loop approaches is the creation of microelectrodes and technologies capable of performing simultaneous stimulation and recording or neural activity.

Currently, bidirectional electrodes that allow stimulation and recording of neural activity at the same time exist, but are limited by the artifacts generated in the recordings by the stimulation (Xu et al., 2018). The detailed description of techniques and materials that allow for the recording of neural activity has been described elsewhere (Stevenson and Kording, 2011; Chen et al., 2017; Hong et al., 2021), but extracellular recordings are the more common type associated with *in vivo* brain recordings. Briefly, electrodes of the order of microns are implanted into the brain and positioned close enough to the neurons of interest to detect the fluctuations in voltage across their membranes. To record from several neurons, a series of microelectrodes can be organized to form a microelectrode array. The main advantage of these microelectrode arrays is that by recording from a number of neurons simultaneously, we can extract more accurately the complex patterns of neuronal activity and get some insights into the information flow (Hong et al., 2021).

Some recent works have led to the development of novel forms of neuromodulation, which are facilitating the ability to manipulate populations of neurons in near real-time. These techniques are based on recording the neural activity around the electrodes and adjusting the electrical stimulation in function of the observed neural activity (closed-loop stimulation). According to the use of the closed-loop approach, these techniques can be divided in device fitting techniques and therapy/efficacy techniques (Table 1).

**TABLE 1:** Examples of closed-loop strategies for neural prostheses.

Aim	Description	Utility	Research/Clinical	References
Epilepsy treatment	Electrical stimulation only when epileptic seizures are detected	Therapy	Clinical	Ranjandish and Schmid, 2020; Farkhondeh Tale Navi et al., 2022
Obsessive-compulsive disorder control	Biomarker-based deep brain stimulation	Therapy	Research	Vissani et al., 2022
Depression control	Biomarker-based deep brain stimulation	Therapy	Research	Scangos et al., 2021
Parkinson’s disease control	Electrical stimulation based on local field potentials (LFP) power	Therapy	Clinical	Little et al., 2013
Parkinson’s disease control	Electrical stimulation based on the phase of hands tremor	Therapy	Research	Cagnan et al., 2017
Fitting of cochlear implants	Fitting of stimulation threshold based on the contraction of stapedius muscle	Fitting	Research	Weiss et al., 2021
Fitting of cochlear implants	Fitting of stimulation threshold based on evoked compound action potential (ECAP)	Fitting	Research	McKay et al., 2013
Fitting of cochlear implants	Fitting of stimulation threshold based on electrically evocated auditory brainstem response (EABR)	Fitting	Research	Guenser et al., 2015
Fitting of cochlear implants	Fitting of stimulation threshold based on cortical auditory evoked potentials (CAEPs)	Fitting	Research	Visram et al., 2015
Spinal cord stimulation for pain therapy	Adjusting stimulation current based on the measured ECAP	Therapy	Clinical	Mekhail et al., 2020
Spinal cord stimulation for motor control	Modulation of gait features through stimulation parameters	Therapy	Research	Wenger et al., 2014
Retinal electrical stimulation for visual restoration	Modulation of electrical stimulation based on retinal ganglion cells response	Therapy	Research	Guo et al., 2018; Spencer et al., 2019; Shah and Chichilnisky, 2020
Fitting of intracortical visual prostheses	Measure the response of V4 neurons to V1 stimulation	Fitting	Research	Chen et al., 2020
Increase efficacy of stimulation in intracortical visual prostheses	Increase efficacy of electrical stimulation in the visual cortex by LFP phase-locked stimulation	Therapy	Research	Allison-Walker et al., 2020
Brain state dependent stimulation in cortical visual prostheses	Look for a brain state in which stimulating is easier to induce visual perception	Therapy	Research	van Vugt et al., 2018

This procedures allow an improved control of some neurological conditions such as epilepsy (Ranjandish and Schmid, 2020; Farkhondeh Tale Navi et al., 2022), and can also be used for better control of obsessive-compulsive disorders and depression (Figee et al., 2022; Visser-Vandewalle et al., 2022). Furthermore, it has been shown that the outcome of brain stimulation to treat Parkinson’s disease can be improved by recording brain activity and stimulating only when the local field potentials collected by electrodes inserted in the subthalamic nucleus exceed a certain threshold (Little et al., 2013), or by associating the brain stimulation to specific phases of patients’ tremor activity (Cagnan et al., 2017). Also, the electrical stimulation of the spinal cord for pain therapy can be adjusted based on the evoked compound action potential (ECAP) (Mekhail et al., 2020), while the movement output in spinal cord stimulation for motor recovery can be controlled in closed-loop changing the stimulation parameters (Wenger et al., 2014).

The above-mentioned approaches can also be applied to the field of sensory prostheses. Thus, the automatic tuning of stimulation thresholds in cochlear implants can be done by measuring the contraction of the stapedius muscle. This muscle contracts to protect the inner ear from very loud sounds (Borg and Zakrisson, 1973) and measuring its contraction provides objective feedback on the loudness of the sound induced by the electrical stimulation (Weiss et al., 2021). Other measures like ECAP (McKay et al., 2013), electrically evocated auditory brainstem response (EABR) (Guenser et al., 2015), cortical auditory evoked potentials (CAEPs) (Visram et al., 2015) have been studied to automatically fit cochlear implants, but none of them reached a clinical application.

In retinal prosthesis, research on closed-loop stimulation have been done to optimize the stimulation parameters to obtain the desired retinal ganglion cells output in response to a given visual input (Guo et al., 2018; Spencer et al., 2019; Shah and Chichilnisky, 2020). The same approach could be applied in cortical visual prosthesis using the activity of cortical neurons instead of retinal ganglion cells. For this to be optimal, a larger part of the visual field should be covered by the electrodes in cortical visual prostheses with respect to the current research devices.

However, even with a smaller covering of the visual field it should be feasible to design and develop similar approaches in the field of cortical visual prosthesis for controlling the timing of stimulation, reducing charge requirements, and fitting the device faster. In this framework, a recent study in monkey visual cortex shows that the activity of neurons in V4 provides direct insight into the efficacy of the stimulation in V1 (Chen et al., 2020). This suggests that neurons in higher visual areas could be used, for example, to estimate and adjust V1 thresholds on hundreds of electrodes. Furthermore, besides adjusting the thresholds, the brain signals collected by the electrodes in the visual cortex could reveal a brain state in which it is easier to induce perception. Some preliminary studies in rats support this point of view and show that it is possible to use the information from the local field potentials (LFPs) as control signals to specify the precise timing of stimulation to reduce charge requirements (Allison-Walker et al., 2020). Moreover, it has been shown in humans that there is a causal relationship between cortical excitation and phosphenes perception so that the phase of pre-stimulus oscillatory activity seems linked to visual perception (Dugue et al., 2011), and other studies suggest that the power spectral density at low frequency (*f* < 30 Hz) contains information about visual perception (Gail et al., 2004). Hence, we can hypothesize that the incorporation of measures of neural activity around stimulating electrodes could be helpful to enhance the effectiveness and safety of any cortical visual prostheses. In addition, we could also incorporate other measures such as linear combinations of brain signals in different bandwidths and even information about the pupil size and eye movements to improve the safety, robustness, and reliability of conscious visual perceptions (van Vugt et al., 2018). Table 1 presents some closed-loop neural stimulation approaches currently used, specifying if they are in clinical or research status.

## Toward personalized closed-loop stimulation in cortical visual prostheses

**FIGURE 1:**
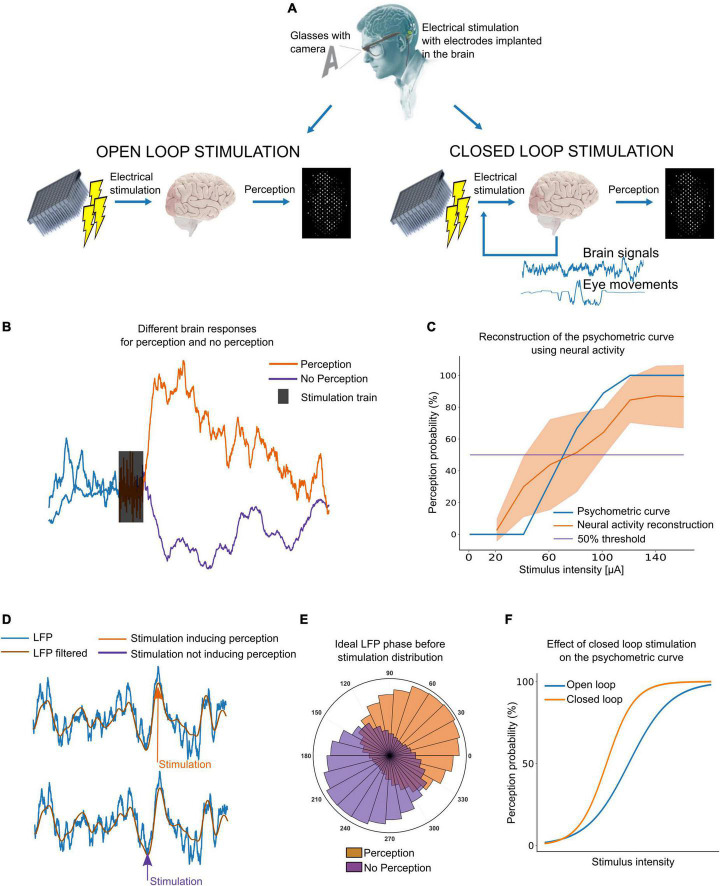
Closed-loop stimulation of the visual cortex. **(A)** Diagram of open-loop versus closed-loop stimulation approaches. **(B)** Example of intracortical brain signals. A clear difference between perception and no perception is needed to adjust the current to induce perception without the user’s feedback. **(C)** Example of a psychometric curve obtained with user’s feedback (blue) and neural signals (orange). **(D)** Local field potentials (LFP) phase dependent response to stimulation. Inducing perception might be easier by stimulating the right LFP phase. **(E)** Ideally, the distribution of LFP phase should be different for perception and no perception. **(F)** Stimulating at the right LFP phase, we could decrease the charge required to reliably evoke phosphenes.

Currently, most cortical visual prostheses are primarily unidirectional or open-loop, passing the visual information from the outside world captured by the image acquisition sensors to the implanted microelectrode arrays. In the future, it is expected that a high number of microelectrodes can be implanted into the brain to provide a functional vision, and such a large number of implanted electrodes may pose several stimulation problems (Fernandez, 2018; Rotermund et al., 2019). Therefore, we have to start reconsidering and improving our methods of cortical stimulation for example with closed-loop approaches (Figure 1A; Rotermund et al., 2019).

It has been estimated that we need at least 625 electrodes implanted in visual areas for reading (although at lower speeds) and navigating through complex visual environments (Cha et al., 1992). However, finding the lowest current thresholds able to induce visual perceptions from each single electrode is a time-consuming procedure that requires the user’s feedback. Moreover, perception thresholds could vary over time, requiring the users to calibrate each electrode many times. As the brain signals surrounding the electrodes contain information about the spread of currents and brain dynamics, we could determine if the currents used are enough to induce perception simply by measuring the brain response to electrical stimulation. For this to be possible, the brain signals during (or after) stimulation should have distinguishable features in case of perception or no perception. Figure 1B shows an example from our ongoing experiments with intracortical microelectrodes in blind volunteers in which the neuronal activity after stimulation increases when the stimulation intensity is enough to induce perception (40 μA in this case). This approach can also be used to construct psychometric curves (relation between stimulus intensity and perception probability) that are practically indistinguishable of the standard psychometric curves using users’ feed-back. Figure 1C shows an example for a single electrode using current intensities from 0 to 140 μA. However, the most robust and reliable features to automatically find perception thresholds still need to be investigated.

On the other hand, there is a need to reduce power consumption and the charge required to elicit reliable phosphenes. In this context, brain activity and other physiological signals could be used to find a brain state in which inducing visual perception is easier, thus decreasing the currents needed to induce the visual perceptions. Using the same stimulation parameters, a given pulse train might produce a visual perception or not according to the LFP phase at which the stimulation is sent (Figures 1D,E). This has been reported in experiments in rats (Allison-Walker et al., 2020) and using non-invasive transcranial magnetic stimulation (TMS) in sighted humans (Dugue et al., 2011), but the feasibility of LFP phase-locked stimulation with intracortical electrodes in humans still remains unexplored. Nevertheless, targeting the right LFP phase before stimulation could allow to reduce charge requirements. Figure 1F shows an example.

## Algorithms for closed-loop stimulation

**FIGURE 2:**
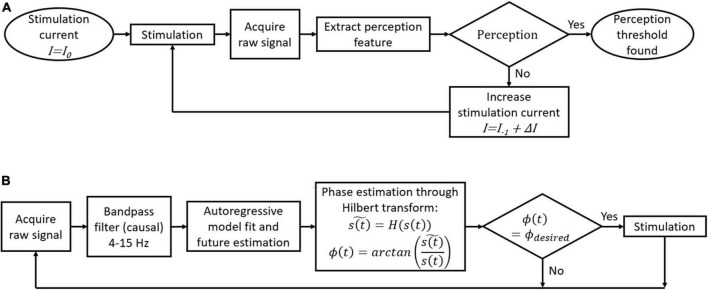
Flowcharts of possible closed-loop algorithms for a cortical visual prosthesis. **(A)** Automatic threshold adjustment for perception. **(B)** Local field potentials (LFP)-phase locked stimulation.

Figure 2 introduces some possible algorithms for closed-loop stimulation in the framework of a cortical visual prosthesis. Briefly, to search for perception thresholds (Figure 2A), a stimulation with an initial current level *I*_0_ is sent from one electrode or a group of electrodes. Then, the brain signals during and after the stimulation (up to 1 s) are recorded and used to extract perception-related features. If perception is detected, the current *I* used to stimulate is set as the perception threshold for that electrode or group of electrodes. If perception is not detected from the extracted features, a new stimulation train is sent with a higher current intensity *I* = *I_–1_* + Δ*I*, where *I_–1_* is the previous current intensity and Δ*I* is the increment of current intensity for each step. The velocity of this algorithm to find perception thresholds depends on the initial current intensity *I*_0_ and on Δ*I* size. Bigger Δ*I* values will speed up the threshold finding at the cost of reducing the precision of the threshold. Furthermore, we can start with *I*_0_ values close to the last perception thresholds to improve processing speed.

To stimulate the desired LFP phase in real time, we can use the approach proposed by Blackwood et al. (2018). First, we have to record 1-second windows of the raw signal and then filter this signal between 4 and 15 Hz. As the phase estimation of the last point in the window data is not accurate without knowing the future behavior of the signal, an autoregressive model is fitted to estimate the future trend. The Hilbert transform is then used to estimate the current phase, and a stimulation train is sent only if the current phase is the desired one (Figure 2B). We have recently tested this algorithm using intracortical signals from the visual cortex of a human blind volunteer at a sampling rate of 30 kHz and we obtained an error of ±20° in the LFP phase estimation (Grani et al., 2022b).

## Discussion

Research on real-time closed-loop neural systems has built upon contributions from neuroscientists, engineers and clinicians, and may prove essential for future cortical visual prostheses, especially when high-number microelectrodes are used. As a result, the next frontier in cortical visual prosthesis may be the development of bidirectional implantable systems with enhanced abilities to modulate and manipulate populations of neurons in real-time. These closed-loop approaches could be able to use information from neural recordings to adjust the optimal stimulation, reduce charge requirements, and improve stimulation performance. However, there are still a number of important issues and challenges to overcome. For example, the stability of signals over time, the influence of movements on signal quality, and the higher energy consumption needed to perform closed-loop stimulation.

Although closed-loop stimulation might increase the safety, performance and usability of cortical visual prostheses, many questions need to be solved before it can be implemented in clinical devices able to continuously record and stimulate from hundreds of electrodes. For instance, the battery of the system needs to last at least for an entire day but adding a real-time brain signal analysis processor to the device would increase the energy consumption. This represents a significant challenge as the sampling frequency often used to get reliable neural signals is 30 kHz. Moreover, in order not to add complexity to the whole device, many signals should be excluded from the closed-loop approach. For example, perception could be inferred from EEG signals in the occipital cortex (Gail et al., 2004), but adding a standard EEG cap to the prosthesis will decrease the overall wearability, and the users might not want to use it on a daily basis.

Intracortical signals captured from the same electrodes used to stimulate cortical areas are the best candidates to build these closed-loop stimulation approaches. However, the signals collected during electrical stimulation are usually corrupted by the stimulation artifacts. Different signal processing techniques and electronic front-end designs can be used to retrieve the signals from stimulation artifacts (Wagenaar and Potter, 2002; Zhou et al., 2018) but these techniques do not work when the amplifiers are saturated. If this is the case, the blanking or exclusion of data during stimulation could be a good option. In addition, a discrimination between perception features and artifacts could be possible assuming that the artifact features increase linearly with the current intensity, while features linked to perception should have a different behavior, starting to increase only after the perception threshold.

On the other hand, as the microelectrodes have to be permanently implanted in the user’s brain, it is important that the signals on which closed-loop stimulation is based are stable over time. Some studies show that the number of reliable spikes captured by intracortical electrodes decreases with time (Sharma et al., 2015), while LFPs are more stable (Grani et al., 2022a). Therefore, closed-loop approaches based on LFP recordings could be more stable over time than approaches based on single neuron spikes and become the basis for future closed-loop approaches.

Datasets using current intensities able to induce perception 50% of the time, could be of great help to get better insight into these issues and help to investigate in which brain state it is easier to induce visual perceptions. Ideally, the LFP phase before stimulation could be significantly different in case of perception and no perception as it is shown in Figure 1E. However, we do not yet know if the same phase of the LFP is valid for all the electrodes. Thus, the neural population around each electrode could determine the preferred LFP phase, which means that to modulate the neural response to stimulation, perhaps we should consider the specific dynamics of every single electrode. In addition, the real-time detection of this parameter can also be associated with uncertainties that reduce the accuracy of phase estimation. Further, although different algorithms have been proposed for continuous phase estimation in real-time (Kim et al., 2016; Blackwood et al., 2018), this locked phase stimulation can also limit the time resolution of the stimulation. Thus, it seems that the frequency of ongoing oscillations is around 10 Hz (Dugue et al., 2011), which means that the preferred phase should appear approximately every 100 ms, limiting to this time the refresh rate of the visual prosthesis. Therefore, all these results must be confirmed in real-life environments, and there is still not enough information about the period of the local field potential that corresponds to maximum excitability nor about how many feedback channels can be reliably provided in parallel.

Another complementary and not mutually exclusive approach could be to reproduce the responses of cortical neurons to different visual stimuli (Guo et al., 2018; Spencer et al., 2019; Shah and Chichilnisky, 2020). Sighted animal models with intracortical electrodes in the visual cortex could be used to obtain visual cortex responses to different visual patterns. Then, knowing the neural activity elicited by a visual stimulus and the neural activity elicited by each electrical stimulation parameter, the stimulation parameters could be shaped to obtain the desired neural activity for a certain visual perception. However, there is no guarantee that eliciting with electrical stimulation the same activity of a natural image in V1 creates the same image perception. In addition, we have to consider that the perception experience is modulated by higher cortical areas (van Vugt et al., 2018) and could also be different in a brain deprived of visual information (Merabet et al., 2007; Fernandez, 2018).

All the progress in neural technologies, neuroscience, electronics, and bioengineering together with increased intelligence in neural systems can help to foster the development of improved custom-tailored devices, which will incorporate advanced closed-loop algorithms for restoring some functional sight to blind people. Therefore, we expect that in the future, closed-loop stimulation will offer more safety, precision, and personalization of cortical visual neuroprostheses approaches.

## Data availability statement

The original contributions presented in this study are included in the article/supplementary material, further inquiries can be directed to the corresponding author.

## Ethics statement

The studies involving human participants were reviewed and approved by Hospital General Universitario de Elche Clinical Research Committee. The patients/participants provided their written informed consent to participate in this study.

## Author contributions

FG, CS-S, AF, and EF contributed to the design, implementation of the research, and writing of the manuscript. All authors contributed to the article and approved the submitted version.
